# The effect of patient body mass index and sex on the magnification factor during pre-operative templating for total hip arthroplasty

**DOI:** 10.1051/sicotj/2023009

**Published:** 2023-05-15

**Authors:** Itay Ashkenazi, Samuel Morgan, Or Shaked, Nimrod Snir, Aviram Gold, Amal Khoury, Shai Shemesh, Yaniv Warschawski

**Affiliations:** 1 Division of Orthopedics, Tel Aviv Sourasky Medical Center, Tel-Aviv, Affiliated to the Sackler Faculty of Medicine, Tel Aviv University 6423906 Tel Aviv Israel; 2 Sackler Faculty of Medicine, Tel Aviv University 6997801 Tel Aviv Israel; 3 Department of Orthopedics, Assuta Ashdod Medical Center, Ashdod, Affiliated with the Ben Gurion Faculty of Medicine 7747629 Beer Sheva Israel

**Keywords:** Total hip arthroplasty, Digital templating, Calibration, King Mark, TraumaCad

## Abstract

*Introduction*: Pre-operative templating prior to hip arthroplasty has traditionally used implant-company-provided acetates, which assumed a magnification factor between 115% and 120%. In recent years, pre-operative planning has been performed with digital calibration devices, in order to calculate the magnification factor. However, these devices are not without their limitations and are not readily available at many institutions. As previous reports suggest a wide range of magnification factors, the determination of an optimal magnification factor is currently unclear. We investigated the relationship between obesity and gender on the magnification factor in order to improve the accuracy of pre-operative templating. *Patients and methods*: Ninety-seven consecutive pre-operative calibrated pelvic radiographs using the KingMark calibration were analyzed using the TraumaCad templating software. The magnification factor calculated by the software was considered the true magnification factor and analysis was made in order to assess the effect of sex and body mass index (BMI) on the magnification factor. A linear regression analysis was utilized to create a predictive model for optimal magnification factor value. *Results*: Magnification factor was significantly affected by sex (male, 120.0% vs. female 121.2%, *p* < 0.01) and by categorized BMI (obese 121.8% vs. non-obese 119.9%, *p* < 0.001). A positive linear association was found between BMI and the magnification factor (*r* = 0.544). The magnification factor was significantly different between the following sub-groups: obese female, non-obese female, obese male, and non-obese male (*p* < 0.001). When applying the model formulated by the linear regression analysis, the calculated magnification factor was within 2% of the true magnification factor for the majority of patients (*n* = 83, 85.6%). *Conclusions*: BMI and gender have a significant effect on the magnification factor. Future determination of the magnification factor should consider the influence of these variables in order to improve the accuracy of pre-operative templating in THA.

## Introduction

Digital templating of pelvic radiographs is of significant importance in the preoperative planning for hip arthroplasty. Such planning helps the surgeon visualize the operation using careful radiographic review. In primary total hip arthroplasty (THA), templating has proven to be helpful in the selection of proper implant size, facilitating the optimization of alignment, correcting leg length discrepancy, and reducing the risk of potential complications [1–3]. In certain instances, templating identifies cases where the required prostheses needed to optimize the function and longevity of the arthroplasty are not routinely stocked in the department inventory, such as extra small femoral components or high-offset. Templating can additionally identify technical issues that may be encountered during surgery such as a narrow femoral canal, dysplastic acetabulum, or poor bone stock.

Pre-operative templating is performed on a standardized radiograph with a known magnification to facilitate the calibration of the implants [4]. Previous reports have recommended a magnification factor of 115–120% [5]. Recently, the advent of a software application with radio-opaque markers to calibrate the digital radiograph has emerged. This technology includes the KingMark (double marker calibration) and the VoyantMark (single marker calibration) calibration, with reports that have substantiated their capability in providing an accurate estimation of implant size for primary THA [6].

Despite their effectiveness, the use of calibration marker devices is not always possible, as they are not readily available in many institutions. These devices are even more limited in trauma patients with hip fractures, due to the difficulties associated with moving the patient for marker positioning. Moreover, in single marker devices, positioning of the marker is complicated by the difficulty of identifying the correct anatomic landmarks by palpation, especially in obese patients [6]. Additionally, patients may find the placement of the marker near their genitals uncomfortable. Double marker calibration devices were introduced to resolve some of the difficulties encountered with the single marker method. KingMark, a double marker device that has been increasingly implemented across departments in recent years, has previously been proposed to be easy to use in patients of all sizes [7]. However, in patients with hip fractures, the use of KingMark is highly inconvenient due to the posterior marker that the patient has to lie on.

This limited ability to use calibration tools and devices in establishing the magnification factor for preoperative planning of patients with hip fractures necessitates the manual selection of the magnification factor. Previous studies have attempted to determine an adequate manual magnification factor for digital radiographs [5, 8]. However, to date, the determination of the optimal magnification factor is still unclear. We believe the wide range of reported magnification factors may reflect differences in patient characteristics, which have not been accounted for. In a previous study [8] White et al. suggested that the magnification factor may be influenced by the size of the patient. Moreover, as a result of differences in pelvic anatomy and fat distribution, likely, sex may also have an impact on the magnification factor.

This study is aimed to further explore the ability to establish an adequate manual magnification factor, by investigating the relationship between patients’ size and sex on the magnification factor, in order to improve the accuracy of pre-operative planning for patients with hip fractures undergoing hip arthroplasty.

## Materials and methods

Institutional research ethics board approval was obtained for this retrospective study (Helsinki number 0192-20).

The study group consisted of patients who underwent primary total hip arthroplasty as treatment for osteoarthritis at our center between January 2015 and January 2016. As part of the pre-operative assessment, all patients underwent a calibrated anterior-posterior pelvic X-ray using the KingMark calibration. X-ray was performed while the patient was lying supine. A radiolucent marker pad was placed behind the pelvis and a marker with radio-opaque balls was placed in front of the pelvis. The beam source was 100 cm from the X-ray plate and positioned 90° relative to the table. Legs were placed in 15° of internal rotation.

The resulting X-rays were uploaded to the TraumaCad software (Voyant Health, Petach-Tikva, Israel) for pre-operative digital templating. Using the KingMark device digital radiograph is calibrated automatically by the TraumaCad software producing a magnification factor. This magnification factor of the automatic calibration was considered the gold standard.

Patients’ data were gathered from their electronic medical records and included baseline patient characteristics such as sex and body mass index (BMI). In accordance with the WHO classification [9], based on their BMI, patients were stratified into two groups: non-obese (BMI < 30 kg/m^2^) and obese (BMI ≥ 30 kg/m^2^).

Statistical analysis was performed using SPSS version 25 (IBM SPSS Statistics, Chicago, IL, USA). Descriptive statistics were applied for age at surgery (years), sex (female/male), and BMI (obese, non-obese). Mean and standard deviations (SD) were calculated for continuous variables and were compared between the groups by Student’s *t*-test. Frequencies and percentages were calculated for nominal variables and were compared between the groups by the Chi-Square test. Linear logistic regression was performed with the magnification factor as the dependent variable, and BMI as the independent variable. Further analysis compared four sub-groups, by sex and BMI: obese female, non-obese female, obese male, and non-obese male. A linear regression analysis was utilized with sex and BMI as the independent variables and the gold standard magnification factor as the dependent variable to create a predictive model for the optimal magnification factor. Validation of this predictive model was made by comparing the calculated magnification factor with the gold standard automatic magnification factor. Differences between cohorts were considered statistically significant for *p* < 0.05.

## Results

Between January 2015 and January 2016, a total of 97 patients underwent digital templating with the use of a double marker calibration of an AP pelvic X-ray as part of the pre-operative assessment for total hip arthroplasty. The mean age at the time of surgery was 66.8 years (SD, 11.8) and 55.7% of the patients were females. The mean BMI was 29.1 (SD, 5.3) with 40.2% of patients being obese and 59.8% being non-obese (Table 1).

**Table 1:** Patient characteristics.

	*n* = 97
Age, mean (SD)	66.8 (11.5)
Sex, *n* (%)	
Female	54 (55.7)
Male	43 (44.3)
BMI, mean (SD)	29.1 (5.3)
BMI Score, *n* (%)	
< 30	58 (59.8)
≥ 30	39 (40.2)
Magnification percent, mean (SD)	120.7 (2.6)

SD – standard deviation, BMI – body mass index.

**Figure 1:**
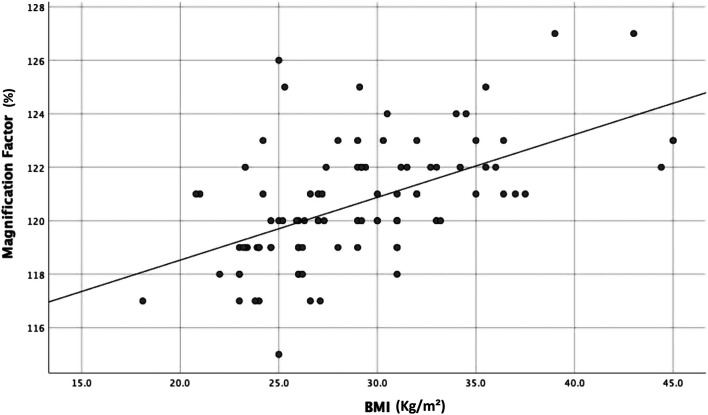
Magnification factor by body mass index (BMI).

Significant differences in the magnification factor were found for sex (male, 120.0% vs. female 121.2%, *p* < 0.01) and BMI category (obese 121.8% vs. non-obese 119.9%, *p* < 0.001) (Table 2). A positive linear correlation (*r* = 0.544) was also noted between BMI and magnification factor (Figure 1).

**Table 2:** Magnification factor by age and by BMI category.

	BMI		
	Obese (*n* = 39)	Non-obese (*n* = 58)	*p*-value
Magnification percent, mean (SD)	119.9 (2.1)	121.8 (2.0)	<0.001
	Sex		
	Male (*n* = 43)	Female (*n* = 54)	*p*-value
Magnification percent, mean (SD)	121.2 (2.3)	120.0 (2.0)	0.01

SD – standard deviation, BMI – body mass index.

Further analysis revealed that the association of BMI and sex to the magnification factor was significantly different between the following sub-groups: obese female, non-obese female, obese male, and non-obese male (Table 3).

**Table 3:** Magnification factor by combined age and BMI category.

*Patients' features*	Number of patients	Mean magnification factor (SD)	*p*-value
Male, BMI < 30	24	119.0 (1.7)	<0.001
Male, BMI > 30	19	121.2 (1.7)	
Female, BMI < 30	34	120.5 (2.2)	
Female, BMI > 30	20	122.3 (2.1)	

SD – standard deviation, BMI – body mass index.

In a linear regression analysis, the patient’s sex (slope −1.03, 95% confidence interval (CI) [−1.77 to −0.29], *p* = 0.007) and patient’s BMI (slope 0.23, 95% CI [0.16 to 0.30], *p* < 0.001) showed a statistically significant correlation with the magnification factor. Based on these findings and the calculated constant (114.47, 95% CI [112.37 to 116.57], *p* < 0.001), we created the following predictive model: Calculated magnification factor = 114.5 + 0.23*(patient’s BMI) – 1*(patient’s sex [women = 0, men = 1]). When applying this predictive model, the calculated magnification factor was identical to the gold standard magnification factor for 28 patients (28.9%), within 1% for 58 patients (59.8%), and within 2% for 83 patients (85.6%).

## Discussion

Digital templating is a routine part of preoperative planning in THA. In situations where calibration markers are not used pre-operatively, there is a need to magnify a pelvic X-ray in order to calibrate it for the templating software. Previous studies have reported a wide range of magnification factors for pelvic images, ranging between 109% and 128% [5, 10]. This wide range of magnification factors may reflect differences in pelvic anatomy and fat distribution that exist across patients with different sex and body sizes. In response to the uncertainty regarding the accurate determination of the magnification factor, we devised the following study to investigate the relationship between patient size and sex on the magnification factor, in order to improve pre-operative templating accuracy in THA.

The main finding of our study was that BMI and sex significantly influenced the magnification factor. When utilizing a predictive model based on these patients’ metrics, the surgeon may predict the magnification factor in the majority of patients. Magill et al. [11] examined 140 post-operative THA and calculated the magnification factor according to the known femoral head and acetabular shell. The authors proposed a fixed magnification factor of 124% for every X-ray. In addition, they found no significant correlation between BMI and magnification. Similarly, Nuria et al. reported no significant difference by gender and no correlation between BMI and the magnification factor, [12] proposing a fixed magnification factor of 113%. The findings of these studies contradict our results. We believe these differences can be explained by the fact that the following studies used a single marker calibration (femoral head, acetabular cup), whereas the calibration in our study was performed with the KingMark device. This double marker device has previously documented superior results, compared to the single marker method for predicting final component sizes after THA [13, 14].

According to the magnification theory, the closer the object is to the X-ray transducer, the greater the magnification that should be used. Therefore, body habitus could likely be a factor that contributes to the final calculated magnification factor. From the findings in our study, we believe that the hips of patients with more fat distribution around their buttocks will be closer to the X-ray beam and consequently, will sustain a larger magnification. The results of our study suggest that size and gender were likely contributing factors to the inconsistent and wide range of magnification factors that have previously been suggested.

Previous studies [15, 16] reported magnification factor inaccuracies in obese patients, using a single calibration marker of known size. The standardized location of the marker (e.g. the greater trochanter) is more difficult to locate in patients that are overweight. Therefore, we believe that in obese patients, a known magnification factor should be used instead of a calibration marker. This may offer an advantage in the ability to accurately predict component sizes in obese patients. Finally, the results of our study suggest that for patients with hip fractures, it may be beneficial to use a predetermined magnification factor which factors in their BMI and gender.

The current study is not without its limitations. The study population could have been larger and was therefore susceptible to a sampling bias. We analyzed two parameters, BMI and gender. It is therefore possible that other patient factors could have influenced the calculated magnification factor. Finally, the study’s retrospective nature presents an additional limitation. Further research addressing these limitations, namely the cohort size, is warranted to improve our understanding of the impact of different patients’ demographics on the magnification factor and to improve clinical applicability.

## Conclusion

In situations where calibration markers are unavailable, or when positioning of the calibration markers is challenging, the determination of a fixed magnification factor should take into consideration the effect of the patient’s sex and BMI. Surgeons should consider the use of this simple predictive model to calculate the appropriate magnification factor.
